# A Harmonized Perspective on Transportation Management in Smart Cities: The Novel IoT-Driven Environment for Road Traffic Modeling

**DOI:** 10.3390/s16111872

**Published:** 2016-11-08

**Authors:** Pavel Masek, Jan Masek, Petr Frantik, Radek Fujdiak, Aleksandr Ometov, Jiri Hosek, Sergey Andreev, Petr Mlynek, Jiri Misurec

**Affiliations:** 1 Department of Telecommunications, Brno University of Technology, 61600 Brno, Czech Republic; fujdiak@feec.vutbr.cz (R.F.); hosek@feec.vutbr.cz (J.H.); mlynek@feec.vutbr.cz (P.M.); misurec@feec.vutbr.cz (J.M.); 2 Institute of Structural Mechanics, Brno University of Technology, 60200 Brno, Czech Republic; masek.j@fce.vutbr.cz (J.M.); frantik.p@fce.vutbr.cz (P.F.); 3 Department of Electronics and Communications Engineering, Tampere University of Technology, 33720 Tampere, Finland; aleksandr.ometov@tut.fi (A.O.); sergey.andreev@tut.fi (S.A.)

**Keywords:** smart city, Internet of Things, embedded devices, genetic algorithm, optimization

## Abstract

The unprecedented growth of today’s cities together with increased population mobility are fueling the avalanche in the numbers of vehicles on the roads. This development led to the new challenges for the traffic management, including the mitigation of road congestion, accidents, and air pollution. Over the last decade, researchers have been focusing their efforts on leveraging the recent advances in sensing, communications, and dynamic adaptive technologies to prepare the deployed road traffic management systems (TMS) for resolving these important challenges in future smart cities. However, the existing solutions may still be insufficient to construct a reliable and secure TMS that is capable of handling the anticipated influx of the population and vehicles in urban areas. Along these lines, this work systematically outlines a perspective on a novel modular environment for traffic modeling, which allows to recreate the examined road networks in their full resemblance. Our developed solution is targeted to incorporate the progress in the Internet of Things (IoT) technologies, where low-power, embedded devices integrate as part of a next-generation TMS. To mimic the real traffic conditions, we recreated and evaluated a practical traffic scenario built after a complex road intersection within a large European city.

## 1. Introduction

Smart city represents a paradigm that is associated with a significant shift of interest towards developing and utilizing multiple innovative communications technologies to make today’s cities more *intelligent* and thus improve people’s quality of life. Being a product of accelerated development employing the advanced information and communications technology (ICT) as well as the knowledge-based economy, smart cities [1] comprise an integration of the Internet, a telecommunications network, a broadcast network, and a wireless broadband network, where the Internet of Things (IoT) becomes a core building block – smart cities include a high degree of ICT integration and a comprehensive application of information resources.

The essential components of urban development for smart cities typically contain (i) smart technology; (ii) smart industry; (iii) smart services; (iv) smart management; and (v) smart life [2,3]. Over the past years, the European Commission (EC) has launched a European Initiative on Smart Cities addressing four pillars of a modern city: buildings, electricity, heating and cooling systems, and transportation [4]. With regards to the latter, the goal of the EC is to identify and support the viable forms of transportation as well as construct intelligent public transit systems based on real-time information, traffic management systems (TMSs) for congestion avoidance, and green applications (e.g., reducing fuel and/or energy consumption) [5].

It is worth mentioning that the number of vehicles that use the existing road network infrastructure in urban areas has seen a tremendous growth [6]. A major consequence of this avalanche is related to the management problems, which range from traffic congestion control to driving safety and environmental impact [7]. Over the recent years, researchers from both industry and academia have been focusing their efforts on exploiting the advances in sensing, communications, and dynamic adaptive technologies to make the deployed road TMSs more efficient with respect to the aforementioned issues within the future smart cities. For traffic management, one of the most critical consequences of road congestion is related to delaying the emergency services (i.e., police, fire, and rescue operations, or medical services). Indeed, human lives, general population safety, and financial risks in case of accidents or criminal attacks strongly depend on the efficiency and travel time of the emergency vehicles. Based on the recent road traffic statistics, an increased number of vehicle crashes was revealed–following the available data, those occur frequently in the areas around congested roads, since the drivers tend to drive faster before or after encountering a traffic jam, with the aim to compensate for the experienced time delays [6].

Today, the world’s largest cities are massively suffering from the traffic congestion despite employing sophisticated mechanisms to reduce it, including the use of TMSs that are advanced congestion control mechanisms. In order to augment the past research efforts aimed at solving the traffic congestion problem (or at least reducing its impact), there is a crucial need to investigate two different types of congestion: (i) recurrent and (ii) nonrecurrent. The *recurrent* congestion happens when a large number of vehicles utilize a certain part of the road network at the repeating time intervals (e.g., morning and afternoon peak hours on weekdays). On the other hand, the *nonrecurrent* congestion mainly follows a random pattern of events, such as traffic accidents (e.g., car crashes or stalled vehicles on the road), bad weather conditions, or special cases including sport events, concerts, etc.

According to the latest information [8], traffic congestion costs billions to the global economy. To illustrate the current situation, the corresponding losses have reached 200 € billion in Europe (2% of the GDP) and $ 101 billion in the USA. Regarding the travel times, the aggregate delays of over 4.8 billion hours were observed due to congestion, while 7.2 billion liters of fuel were wasted worldwide. These statistics provide a clear indication of the devastating effects that road congestion has on people, companies, and society. Unfortunately, the existing TMSs are still unable to provide detailed and accurate information to enable fine-grained and timely monitoring and management of the road traffic [9,10].

The underlying reasons include the (i) lack of granular data collection; (ii) inability to aggregate the needed volume of data (see e.g., the Big Data paradigm [11]); and (iii) absence of the adequate management systems that report on the actual state of the road network [12]. This leads to an overall inability to effectively monitor and manage the traffic, which negatively affects road safety and fuel consumption, as well as causes increased gas emissions [8]. Presently, the implemented solutions utilized by the existing TMSs to manage the road traffic (e.g., after an accident or during the rush hours) are e.g., adapting traffic lights intervals (cycles) or dynamically closing road lanes and intersections. However, these solutions have rather limited efficiency when increasing numbers of vehicles [13] are using the road infrastructure of a fixed capacity—since efficiency of discrete solutions is limited. Therefore, new implementations and mechanisms are being proposed by the research community to improve the traffic management systems. Authors in [14] present the stochastic traffic environment utilizing a genetic algorithm that optimizes the traffic signal timing (based on a characteristic non-linear function that uses two major performance measures: (i) queuing lengths; and (ii) vehicular waiting times). In [15], the adaptive traffic control system (ATAK) is discussed, which is able to optimize traffic signal timings on signalized road network in real time. Further, simulation of proposed genetic algorithm (for management of the traffic signal timing plan) is elaborated in [16]. Another approach, where the technology able to count the number of vehicles using the video image detection is described in [17]. Aiming to integrate the simulator and the created prototype, authors in [18] introduce a solution for verification of the proposed smart traffic optimization system—the communication between vehicles was realized using the ZigBee-based technology.

**Figure 1:**
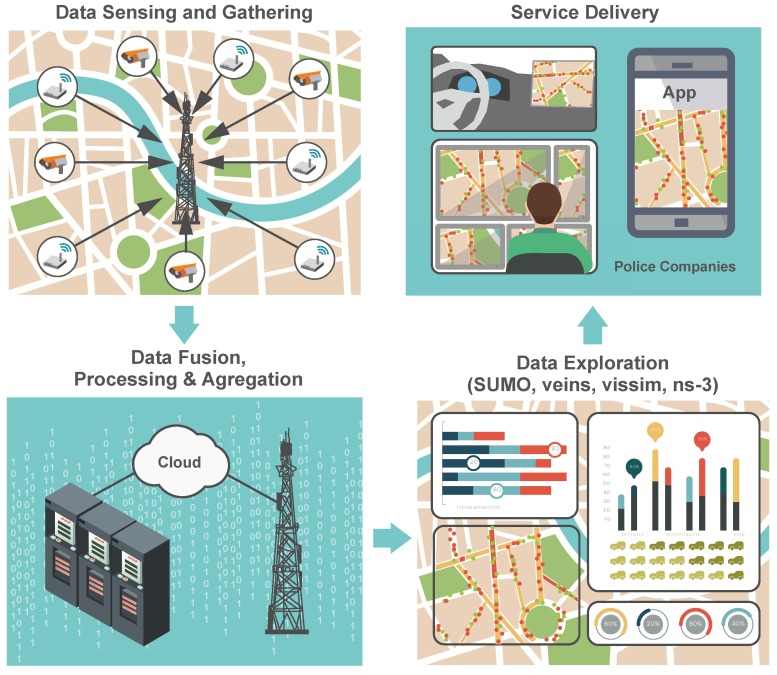
Communications chain of data feeds in smart transportation (parts of the traffic management system).

In this paper, we provide a systematic study of the promising solutions to be employed by the existing and future TMSs, by specifically investigating the different phases of a modern traffic management system in a smart city environment, see Figure 1. In particular, this work discusses two important phases. The first phase is named data sensing and gathering and utilizes heterogeneous road monitoring equipment that measures and communicates the traffic parameters (e.g., traffic volume, vehicle speed, road segment occupancy, etc.) to a traffic management entity (TME). Furthermore, this paper highlights the advantages of various related improvements, such as mobile sensing to improve the efficiency and accuracy of a TMS. Based on the pattern of the collected information, the second phase introduces our created environment for modular traffic modeling that allows a user to construct a road network under investigation in its full resemblance. Describing the topography of the problem at hand together with the traffic control signalization features, the resulting model exhibits qualitatively and quantitatively appropriate behavior. Our proposed environment is further examined on several low-power and low-energy (embedded) devices with respect to meeting the IoT goals, where power-constrained equipment will take over as computation units located throughout a smart city [19].

The rest of this paper is organized as follows. In the subsequent section, we offer an overview of the future TMSs by highlighting their important conceptual phases and design stages. Further, Section 3 is devoted to addressing the data sensing and gathering (DSG) phase featuring a description of various communications technologies utilized for road traffic monitoring. It also discusses alternative technologies that may improve the quality and the accuracy of the received (collected) data. Then, in Section 4, we outline our constructed environment for modular traffic modeling. Later, in Section 5, our attention is shifted to the performance evaluation of two characteristic experimental scenarios (following the real traffic patterns within the city of Brno, Czech Republic). Here, different embedded devices, such as (i) Intel^®^ Galileo; (ii) Edison; and (iii) Raspberry Pi, were employed. In the concluding Section 6, the lessons learned in the course of developing our proposed modular environment for traffic modeling are summarized.

## 2. Reviewing Road Traffic Management Systems

Today, the traffic management systems offer capabilities that can be utilized to reduce the road congestion, improve the response times in case of car accidents (related to delaying the emergency services), as well as enhance the travel experience of commuters. Typically, the TMS logic comprises four complementary phases, as depicted in Figure 1. The key building block of the represented chain is data sensing and gathering functionality, in which heterogeneous road monitoring equipment [20] measures the important traffic-related parameters (e.g., traffic volume; speed; and occupancy of the road segments) over certain time intervals. Further, the measured data is forwarded to the central TME; the detected events are immediately reported over the deployed wireless communications networks (e.g., cellular systems). Then, the obtained data feeds are processed (aggregated) in the second block known as the data fusion, processing, and aggregation (DFPA). The following block, named the data exploitation (DE), processes the knowledge from DFPA and computes the (i) optimal routes; (ii) short-term traffic forecast; and (iii) supplementary road traffic statistics. The final block, termed the service delivery (SD), distributes the resultant knowledge to the end users/commuters (e.g., drivers, private companies, emergency services, etc.).

It is important to note that the utilization of features delivered by the TMS is not limited to drivers and authorities, but may also significantly contribute to the economic growth, safety of citizens, and national security. Currently, the deployed systems and technologies are still unable to acquire accurate traffic parameters and can miss real-time reports of accidents that occur along the road networks, especially in developing regions. Operating more sophisticated equipment to ensure highly-accurate assessment of traffic flows and timely detection of emergency situations may seem to be a preferred solution. However, this choice remains limited due to constraints in financial resources available to support the needed dense deployment and maintenance of such costly equipment, which may lead to a lack of flexibility [9]. Therefore, alternative cost-efficient and agile solutions are needed to offer better management of road traffic.

To overcome some of the aforementioned constraints, modern TMSs aim at adopting innovative technical approaches to exploit more advanced solutions and thus monitor the evolving road networks more closely. In particular, this allows for exploiting the short-term predictions based on current traffic volumes to identify the impeding bottlenecks and make more informed decisions on how to reroute traffic, change lane priorities, and modify traffic lights sequences most appropriately. Further, contemporary traffic management services need to also provide visual tools for displaying the real-time traffic situation related to the (i) location of traffic jams; (ii) accidents; (iii) congestion levels for each of the road network segments; and (iv) estimated travel time across the road network. Using this information, responsible authorities will have a better real-time perspective on the road network and might in turn conduct more adequate traffic management as well as achieve more efficient reactions in case of emergency and road accidents.

**Figure 2:**
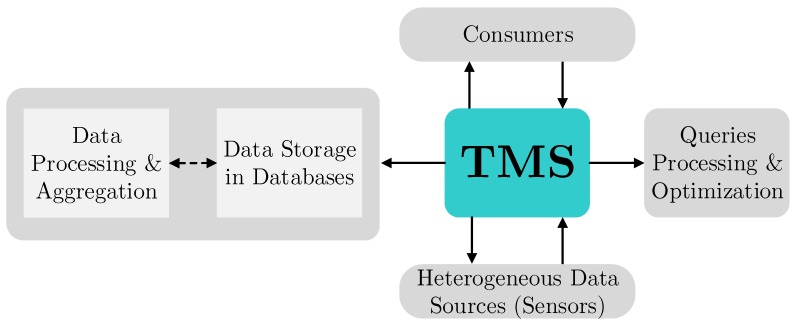
Overall architecture of a modern traffic management system.

To summarize the requirements on the traffic management services in future smart cities, the following aspects need to be taken into account: (i) ability to ensure higher accuracy in estimating road conditions; (ii) better efficiency in resolving emergency situations (as compared to the existing TMSs); (iii) effective management of traffic across diverse road infrastructure; (iv) providing real-time traffic simulation and visualization to assist national authorities in managing the road infrastructure and in route planning; (v) ensuring a simple integration of the existing systems and implementing new technologies—the architectural overview of a contemporary TMS is summarized in Figure 2.

The main components of the TMS aim to deliver the collected traffic related data to the intended end user (e.g., police, drivers, etc.). The core of the TMS collects the road information from heterogeneous data sources (e.g., sensors and other measuring devices) according to the end user needs and specific requirements. Thus collected data feeds are then aggregated and stored in a unified format within one or several databases. Next, upon the reception of a consumer request, the core system processes this request and extracts the applicable data from an appropriate database. Then, the requested information is sent back to the intended end user, possibly tailored for a particular purpose (e.g., analysis and statistics, decision making, etc.).

## 3. Traffic Data Sensing and Gathering

As discussed in the previous sections, data sensing and gathering focuses on scalable collection of road information from a large number of heterogeneous sources. The presently deployed systems in use by the traffic management companies collect data across a variety of formats, time scales, and degrees of granularity. This is because of the fact that said systems have been deployed at different time periods, all having limited functionality for the purposes of the data exchange between them. In contrast, the emerging TMSs are expected to employ a number of traffic related information collection principles as demanded by the city authorities in order to identify gaps where new communications technologies and systems could be deployed to improve the (i) accuracy; (ii) timeliness; and (iii) cost efficiency of data gathering.

Following the aforementioned requirements, the current trends in development of TMSs comprise the use of advanced communications and sensing technologies (e.g., wireless sensor networks (WSNs), cellular networks, mobile sensing, and social media feeds) as potential solutions to extend the limitations of the existing systems in the upcoming era of Smart Cities. As the main wireless technology utilized for data sensing and gathering on the road networks, embedded devices in the role of small sensors are being deployed ubiquitously—they can be mounted on the vehicles, at the roadside units (RSUs), or even under the pavement to sense and report the unexpected events.

In case of embedded in-vehicle sensors, the parameters related to the car operations are monitored and measured. Further, the data is disseminated to the nearby vehicles and/or the RSUs. In case of embedded devices, sensors are primarily used for measuring the speed of passing vehicles, the traffic volumes, or other parameters of the environment. A modern TMS needs to therefore concentrate on leveraging the innovative solutions that enable collection of data from a specific region of interest under particular time constraints while minimizing the cost and spectrum usage as well as maximizing the system utilization.

### 3.1. Wireless Sensor Networks

**Figure 3:**
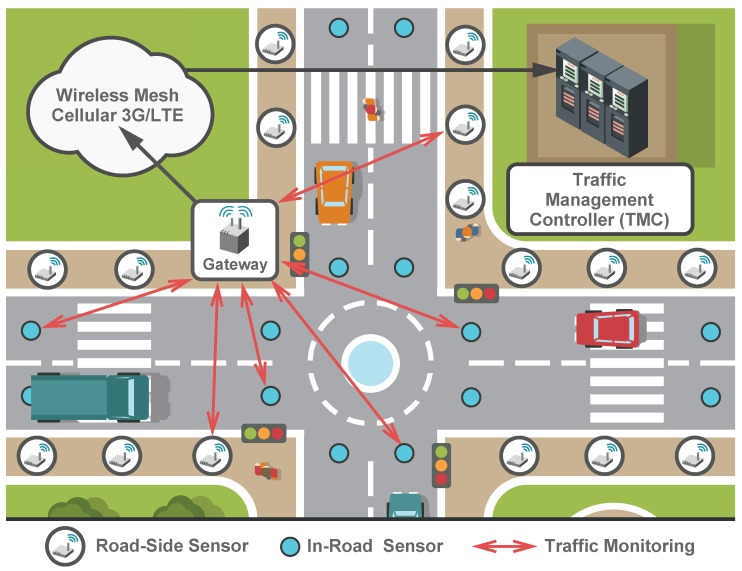
Communications chain of data feeds in smart transportation.

Offering high efficiency and accuracy in tracking different events, wireless sensors have already been deployed widely in various environments for the purposes of data collection and monitoring [21,22,23,24,25]. The examples of such implementations are the real-time control of traffic lights [26,27,28] and their adaptation according to the road congestion levels [29] as well as urban parking management [30]. However, the deployment of WSNs in the road environment faces further challenges in addition to the well-known issues [31]. These drawbacks require a careful choice of an appropriate routing protocol. Among said challenges, there is a need to offer fast and reliable Medium Access Control (MAC) functionality and data forwarding mechanisms [32]. An example of the WSN deployment for traffic monitoring is illustrated in Figure 3.

In this context, it is worth noting that the anticipated wide and dense networks of wireless sensors on the roads require utilization of data aggregation techniques to combat the high redundancy and correlation within the transmitted information. To reduce this data traffic redundancy, the optimized placement of wireless sensors alongside roads should be investigated. In addition, the tradeoff between the number of deployed sensors and the road events detection accuracy needs to be characterized.

### 3.2. Machine-to-Machine Communications

Machine-to-Machine (M2M) communications technology is often named to be a promising enabler for reliable and fast data sensing and gathering [33,34]. It has attracted focused attention from both academia and industry to advance the data collection applications in different environments [35]. Following the recent forecasts by the leading telecommunications companies [36,37,38,39], an unprecedented growth is projected for the M2M devices over the following decade—billions of nodes will be able to utilize the communications channels offered by the M2M technology.

Currently, around 5 billion wireless devices are connected to various WSNs and there are forecasts telling that this number will grow to 50 billion of connected objects by the end of this decade [40]. In M2M communications, sensors (measuring devices) collect the required data and send it over the wireless links (3G, 4G (Long Term Evolution, LTE)) to a remote central server for further processing [41]. The ability of the M2M devices to avoid multihop transmission makes data delivery faster and more reliable, thus becoming a key advantage comparing to the legacy WSNs. Furthermore, the M2M technology will significantly enhance the accuracy of data sensing and gathering as well as lead to a more flexible deployment of sensors and embedded devices.

We continue by reviewing some of the available technologies for M2M communications. M2M connectivity over LTE networks is expected to become a key consideration in the future TMSs. The M2M devices equipped with LTE transceivers are capable of communicating with a remote server (that aggregates and collects the received data) in a reliable, fast, and thus extremely efficient manner. Furthermore, the native support of Quality of Service (QoS) classes enables effective collection of prioritized data from multiple sources as well as guarantees the needed QoS levels for each of the data streams. However, deploying massive M2M nodes as an alternative to the currently installed WSNs will incur additional costs related to the use of cellular (3G, 4G, and 5G (emerging)) networks [42,43]. Therefore, the attractive ICT choices and associated frequency bands are being reviewed, see Table 1 where a description of Low-Power Wide-Area (LPWA) technologies is given.

Fueled by the large numbers of M2M devices (in-vehicle sensors), Vehicle-to-Vehicle (V2V) and Vehicle-to-Infrastructure (V2I) communications are expected to play an important role in the development of TMSs across future smart cities [45]. To ensure the required levels of efficiency for this new type of connectivity, the reliability of a WAVE system together with the IEEE 802.11p MAC protocol [46,47] has to be taken into account in relation to the information dissemination (i.e., routing protocols). Generally, the routing protocols utilize either the (i) road network map; (ii) vehicle mobility model; (iii) both of them; or (iv) neither of them to determine an end-to-end route between the source and the destination vehicles (and sensors therein). A summary on the routing protocols for V2V/V2I communications is offered in Table 2. As shown in Figure 3, the routing protocols transfer data from the vehicles via the sensors to the gateway (often named the Machine-Type Communication Gateway (MTCG) [48]). At the gateway side, the data is sent (usually employing cellular technology, such as 3G or 4G (LTE)) to a remote traffic management controller (TMC) [49]. Then, the relevant TMC information can be used for traffic modeling as described in the following Section 4.

### 3.3. Mobile Sensing

In context of the overall discussion in this section, mobile sensing by utilizing the mobile terminals is expected to enable the fast detection of relevant events on the roads as well as increase the accuracy of the traffic condition monitoring. Following the recent findings in [50,51,52], mobile crowd-sensing systems using modern smartphones have been successfully applied to provide with a more accurate real-time road traffic information over large areas (e.g., enabling precise localization of vehicles, improving end user/commuter travel experience, etc.).

A key requirement for the proliferation of mobile sensing technology is the voluntary participation by the end users. On the other hand, to protect this user-provided data, commuters might demand high levels of privacy and stringent security guarantees before they agree to participate in such data collection. These considerations become the main hurdles and have to be carefully addressed to motivate a large number of people to join mobile sensing applications. The indicated issues may be solved by the following: (i) trust management of mobile sensing data sources; (ii) privacy preservation of user device owners; and (iii) utilization of robust authentication techniques [53].

### 3.4. Social Media Feeds

Closely connected with the notion of smart cities, social media feeds (e.g., Twitter, Facebook, Google+, Waze) can play a crucial role in improving the accuracy of the road traffic information provided by the conventional monitoring equipment (e.g., road sensors, induction loops, etc.) [54,55]. As an example, revealing the possible causes of an unexpected increase in the congestion levels (e.g., car accidents, road works, or mass political and social events), social media sources may help achieve a more appropriate reaction from the side of the responsible authorities to mitigate the effects of these situations [56].

At the same time, it is important to note that there is a need to verify the accuracy of the provided data to prevent from exploiting the unreliable sources of information. To this end, several research groups addressed the design of powerful security and privacy preservation solutions [57,58,59,60,61,62,63].

## 4. Proposed Model Description

To advance the concept of constructing TMSs featuring the M2M devices for future smart cities (as discussed in the previous sections), we describe our proposed framework for traffic modeling, which is capable of running on top of low-power equipment. The presented environment for modular traffic modeling allows the user to easily construct the examined road network in its full resemblance. Describing the topography of the problem at hand as well as the traffic control signalization features, our model exhibits both qualitatively and quantitatively appropriate behavior.

The generality of our proposed implementation enables such features as priority right/left conditions, priority to crossed lanes while turning on a junction, one-way routes, roundabout junctions, automatic synchronization of selected traffic signals, and many more.

### 4.1. Basic Components of the Model

The basic topography of each created model is based on the dedicated nodes. Each of the nodes has planar coordinates of an important point in the described model, such as the direction change, splitting or merging the lanes, the origin of a separate turning lane, or just making the topography of the route more precise. Two nodes are typically connected with a lane object, which keeps the properties of its length, direction, the maximum vehicle speed, vehicle capacity until congested, and vehicles traveling on the route.

The topography construction in our model is performed either manually in the source code or by using already developed methods. The first possible way of simplifying the input process is to use the implemented text reader for inserting the planar coordinates from a *.txt or *.csv file.

An advanced input method is being developed as well. For a convenient utilization of the resultant application, we are currently implementing a procedure that imports all of the topographic entities for our model (i.e., planar coordinates of the nodes, node objects, and lane objects) from a file in a *.dwf format.

### 4.2. Traffic Signals and Their Synchronizations

A lane to which a signal object is assigned becomes a “signalizable” lane object. A signal object keeps information on the duration of each signal period and its phase in seconds. In practice, it means that the variables of a signal object are integer values of durations of signal periods: green, orange-to-red, orange-to-green, red, and duration of phase shift of the signalization. A signalizable lane also acquires an ability to maintain vehicles, which have reached the end of the route and are waiting for a possibility to drive away.

Two or more signal objects can be merged into a signal “bunch” object, which allows to simultaneously bond the timing and phasing of all the contained signals within, i.e., multiple signalizable lanes are operated by an identical signal. This possibility is utilized when creating any junction where traffic in opposite or other directions shall proceed simultaneously.

Further, a bunch list object can be created when merging multiple signal bunches. A bunch list maintains a possibility of synchronizing phases of the contained signal bunches, ensuring that traffic flows will not collide.

### 4.3. Priority Conditions

As explained so far, the model lacks an ability to create scenarios such as priority right/left conditions, giving it to crossed lines while turning on a junction, or conditions required for operating a roundabout junction. For this purpose, the “crossed” lane object has been developed.

The “crossed” property may be assigned to any lane type object, along with the specification of another lane, which traffic is considered superior to the said “crossed” lane. Any lane can comprise multiple “crossed” lanes. Then, the traffic transferred by this lane is only allowed to proceed if all the “crossed” lanes are empty and its signal is green, if assigned.

**Figure 4:**
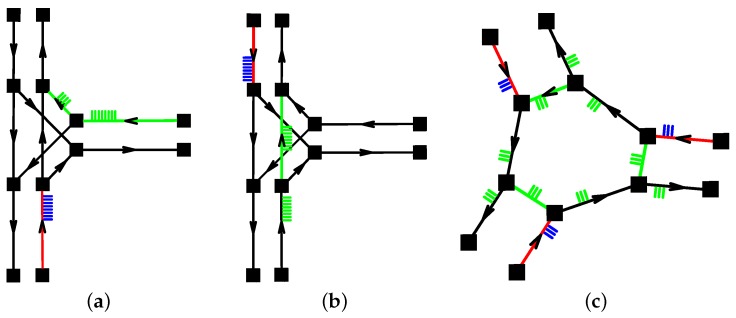
Crossing lane condition features. (**a**) Priority right; (**b**) Crossing a lane when turning left; (**c**) Roundabout priority left.

Using the crossed lane feature in the model, it is possible to construct all the discussed scenarios in a systematic manner. The use of the crossed lane feature is illustrated in Figure 4. On the very left, the utilization as a priority right condition is shown, see Figure 4a. The cars traveling on the red lane are allowed to leave it (if these intend to continue straight ahead) only if there are no cars traveling on the green lanes (assigned as crossed lanes to said lane). The example depicted in the middle, see Figure 4b, illustrates the same condition as one for modeling priority to vehicles on the crossed lane when turning left.

On the far right, see Figure 4c, the last mentioned example captures the construction of a roundabout. In this case, cars on the red lanes give priority to cars traveling on the green lanes, since the European legislation dictates priority left.

Further, the priority conditions shall be applied only to sections of the road which, when conducting traffic, generate a possibility of an accident. There is, however, no reason to give priority rights to vehicles which are too far to become a threat.

### 4.4. Characterizing a Congestion

The ability to characterize a congestion emerges due to two main properties of our model. First, each lane is considered to be of a finite length. With finite speeds of traveling vehicles of non-zero lengths, a lane has a finite capacity and could thus become congested. As soon as the capacity of a lane is reached, arriving cars cannot enter said lane and begin to stack in the preceding lanes until when the route is open again.

A significant congestion is typically caused by the signalization. As the signal of a signalizable lane turns red, vehicles that have reached the end of the lane begin stacking on said lane. When the capacity of the signalizable lane is reached, the preceding lanes start to eventually congest. As soon as it is possible to drive further, the cars start their motion from their actual positions on the road.

### 4.5. Traffic Generation and Routing

Once the desired topography and signalization of the road traffic model are created and synchronized, the traffic generation process may begin.

First, the routing procedure reads all the lane objects from a model, including their directions, lengths, and maximum speeds. After the user specifies the required density as well as the entering and exiting lane of each traffic flow, the implemented Dijkstra’s algorithm [81] manages the idealized routing of every single traffic flow.

The density of the traffic flow itself can be generated as a constant homogeneous process or as a function of time. Its stochastic description can be applied as well.

### 4.6. Target Problem and Objective Function

Generally, an objective function (or a norm) refers to a construction that serves for evaluating the performance of an optimization result. The users may choose their own desired properties of an idealized road traffic flow. Examples include [82] the lowest average travel time, the minimal queuing time, the lowest car density, the maximal average vehicle speed, the number of vehicles transferred, etc. An important challenge is to identify such a setup of signal durations and phasing, which leads to the desired idealized result (assuming that the traffic flow is known).

Mathematically, the objective function is a formulation that takes a vector of functions at its input and returns a single scalar value. Let us consider a functional *f* of the input vector x(t) as f(x,t). Then, if all the state variables and their mutual relations are known as well as the functional is analytically describable and differentiable for all the state variables, the minimum of the functional would be reached under the following condition:

(1) $$\delta \int_{t0}^{t1}f\left(x,t\right)=0,$$

where *δ* is the variation of the functional f(x,t). In other words, derivatives of a function with respect to all state variables and time are zero:

(2) $$\frac{\partial f(x,t)}{\partial x_{i}}=0,\;\;\;\;\;\;\frac{df(x,t)}{dt}=0.$$

However, this is not the case for the problem at hand, as it is infeasible to describe the relations between all the state variables and their impact on the target solution. We argue that the discussed problem is that of the NP-hard nature [83] and the mentioned solution principle cannot be applied. Moreover, minding the dimensions of the configuration space, any combinatorial approach would be effectively non-applicable either.

In the scenarios presented below, we consider the average travel time $t_{\mathrm{avg}}$ as the objective function:

(3) $$t_{\mathrm{avg}}=\frac{1}{N_{\mathrm{vehicles}}}\sum_{i=0}^{N_{\mathrm{vehicles}}-1}\left(t_{\mathrm{out},i}-t_{\mathrm{in},i}\right)\to \min,$$

where $N_{\mathrm{vehicles}}$ is the count of all the vehicles transferred, $t_{\mathrm{in},i}$ is the entering time of a particular vehicle, and $t_{\mathrm{out},i}$ is its exiting time. One could also consider using the total sum of vehicle travel times as the norm, since dividing by a constant number of traveling vehicles does not affect the solution point.

### 4.7. Solution Process

Targeting the embedded devices with very limited resources, an efficient solution method has been developed. The road traffic modeling is performed in a discrete dynamic way, but does not require a fine time discretization step to maintain stable behavior. Our solution method operates with a fully adaptive time step. The reason being is that all the behavior within the traffic model, such as signal changes and car movements (entering and leaving the lanes, stacking in a queue, etc.), is implemented in the event-driven fashion. Hence, the time step of the simulation dynamically changes in an adaptive manner and according to the processes inside the model. It is worth noting that such an adaptive time discretization exhibits adequate stability levels in our solution.

### 4.8. Optimization Process

The search for the preferred setup and synchronization of the traffic signals subject to the given traffic flow density and direction is performed by utilizing a genetic algorithm. Despite the reasonable performance of our genetic algorithm implementation, other heuristic methods can be applied as well, including, e.g., simulated annealing [84].

Before the actual optimization task can be executed, the traffic model needs to be assembled. The latter means loading the coordinates of nodes and connecting these with lanes. Further, signals are assigned to their respective lanes. The model is completed by merging signals into signal bunches, if their timing and phasing has to be concurrent. Finally, bunch lists have to be created and filled with needed signal bunches, synchronization of which is maintained by every bunch list.

The last phase before the optimization is started includes generating the traffic flow properties. This means characterizing each traffic flow by its enter lanes (on which the flow enters the model), exit lanes (the destination of the flow), and its intensity (vehicles generated per second). All the described traffic flows are then routed using the implemented Dijkstra’s algorithm. At this point, everything needed is set and the model is ready to conduct the traffic scenario.

The basic part of the implemented genetic algorithm is an object named genome. The genome here represents a unique setup of traffic signals, corresponding to durations of signal periods and synchronization obtained from bunch lists. Multiple genomes form one generation of a population.

Generally speaking, a fairly standard genetic algorithm may be run. For each genome in the population, a dynamic traffic simulation is executed utilizing the very specific signal properties carried by said genome. During each simulation, information about the transferred traffic is collected and the value of the objective function is calculated and stored in the properties of a particular genome. After every simulation, the current traffic is cleared from the model and the traffic generation is reset. At the very beginning of the optimization process, the initial population is created utilizing genomes with randomized values of their signal properties.

After the simulations for the entire population are completed, a genome selection process is conducted. According to the desired selection ratio, a certain part of the population is removed in the process of tournament selection [85]. This selection is based on a comparison of the objective function values for two random genomes. The genome with a worse performance is then removed. It is also possible (and efficient) to keep a certain possibility that the less suitable genome wins the tournament. This phenomenon leads to a more varied structure of the population and allows to explore a wider array of possible global minima.

The resulting gap in the population shall be subsequently filled with new genomes. The creation of new genomes comprises two combined methods. The former is the creation of an exact clone for one of the remaining genomes and a subsequent mutation of properties for the contained signals (i.e., the duration and phasing of traffic signals). The latter is known as the interbreeding process. A random couple of remaining genomes is selected and the objective function values of both genomes are compared. Then, a brand new genome is created as blank and inherits signal properties from its “parents” proportionally according to the ratio of their objective function values. The overall ratio between the reproduction by mutation and by interbreeding can be adjusted as desired.

As soon as the population is restored back to its maximum size, the generation of genomes experiences the optimization process similar to that for their ancestors. Said optimization process is briefly sketched by the following Algorithm 1.
**Algorithm 1** Optimization via a genetic algorithm (GA) **Input:** Nodes, Lanes, Signals /* Model creation */ Assignment of Nodes to Lanes Assignment of Signals to selected Lanes Merging selected Signals into SignalBunches Merging selected SignalBunches into BunchLists /* Routing and car generation */ Parameters of traffic flows (enterLanes, exitLanes, intensities) Generating traffic flows (parameters, duration) /* GA optimization */ **for** gen = 0; gen < maxGenerations; gen ++ **do**  **for** genome = 0; genome < popSize; genome ++ **do**   Load genomes specific signalBunches; Synchronize BunchLists   Load traffic   Simulate traffic model   Calculate objectiveFunction value   Clear traffic  **end**
**for**  Sort population  TournamentSelection (ts ratio)  Reproduce population (mutate ratio) **end**
**for** **Output:** Genome with the best performance (signal parameters) from the last generation.

## 5. Experimental Results

### 5.1. Classical T-Shaped Junction Scenario

**Figure 5:**
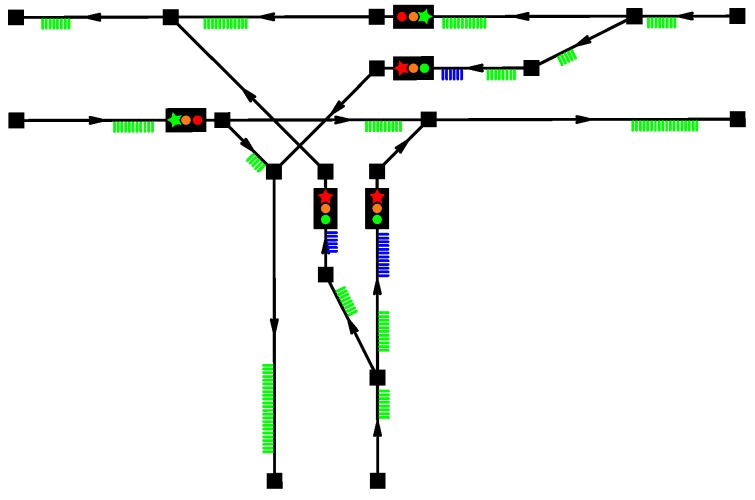
First example: T-shape junction.

Our first presented example illustrates the optimization problem for a T-shaped junction. The topography of the model as well as the signalization and the road traffic flow are shown in Figure 5.

One can clearly observe the discussed structure of each model – the nodes connected by lane objects. For the specified lanes the signals are assigned, as discussed above. Then, the signals with the same expected timing are merged into the signal bunches, and all the signal bunches are then maintained and synchronized by a bunch list. The transferred traffic is displayed as well: the vehicles that are in motion on a lane (that is, handled by the respective lane’s motion queue) are rendered in green. The vehicles that have reached the end of a lane are rendered in blue, since they are waiting for a possibility to leave their respective lane.

### 5.2. Real-Life Scenario

**Figure 6:**
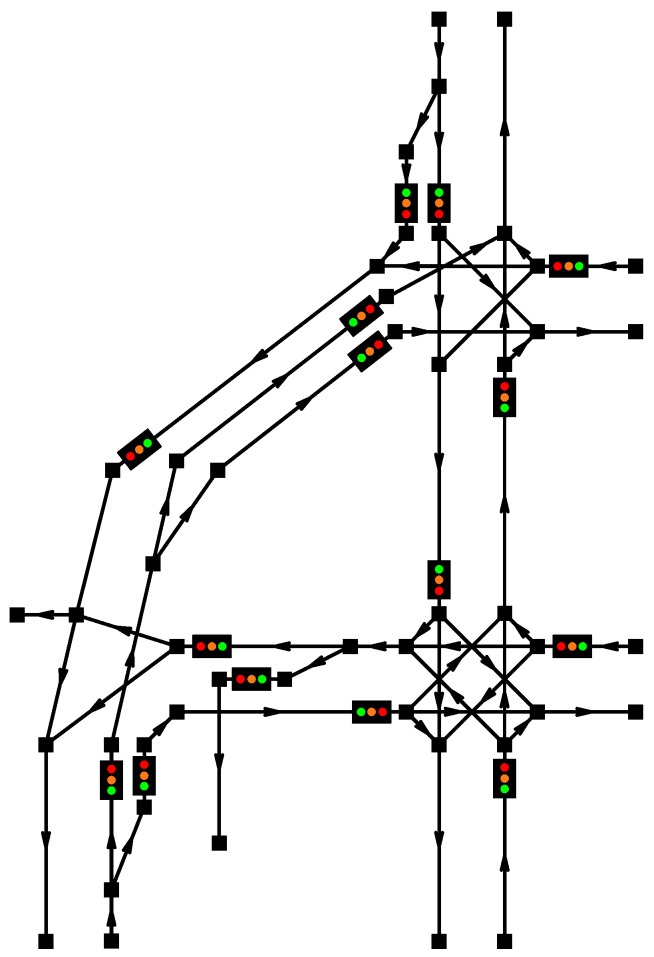
Second example: real traffic scenario, Brno, Czech Republic.

**Figure 7:**
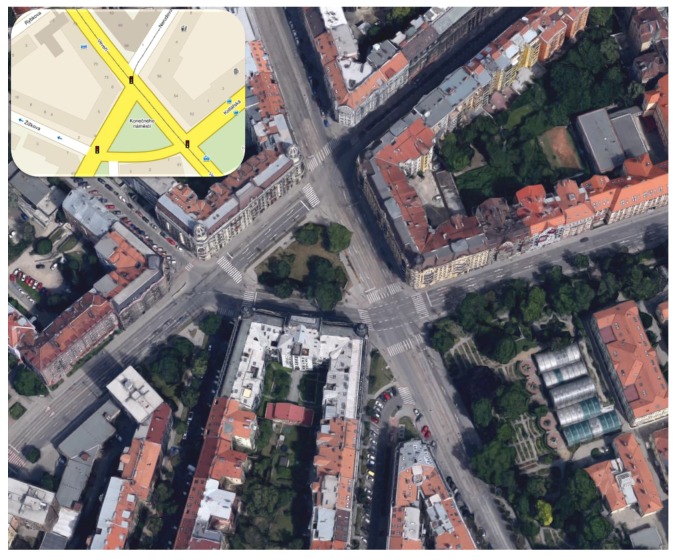
Second example: map view of the real traffic scenario, Brno, Czech Republic (Location: Konecneho square).

Our second presented example, see Figure 6, describes an actual city traffic scenario as deployed in Brno, Czech Republic [86]. This scenario was chosen due to its high complexity, which allows to utilize most of the implemented features in the real-life conditions [87]; the map view of the real traffic scenario is depicted in Figure 7.

### 5.3. Stability and Convergence of the GA Method

The following subsection introduces and discusses the experienced behavior of the optimization method as well as studies its dependence on the particular parameters. All of the collected results represent the average figures obtained by running 50 simulations with random initial parameters of traffic signals.

**Figure 8:**
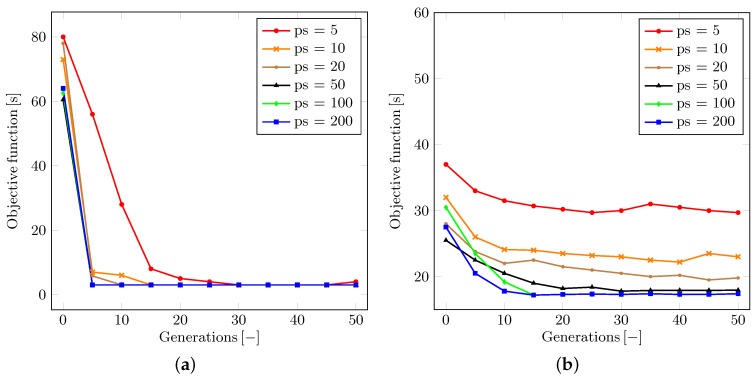
Optimization results with respect to the population size. (**a**) Classical T-shaped junction scenario; (**b**) Real-life scenario.

The first discussed topic is the dependence of the population size (the number of genomes) within each generation. Let us denote the population size parameter as ps. This being said, Figure 8 illustrates the development of the appropriate objective function for each generation in the optimization of both scenarios, respectively. One can conclude that an overly small population may result in unstable or even divergent optimization process. The minimal population size needed for the asymptotically convergent solution clearly grows with the complexity of the problem at hand. This property has a potential to be crucial for the increase in the time consumption. However, it does not disqualify the use of the proposed model considering the time requirements of each simulation.

**Figure 9:**
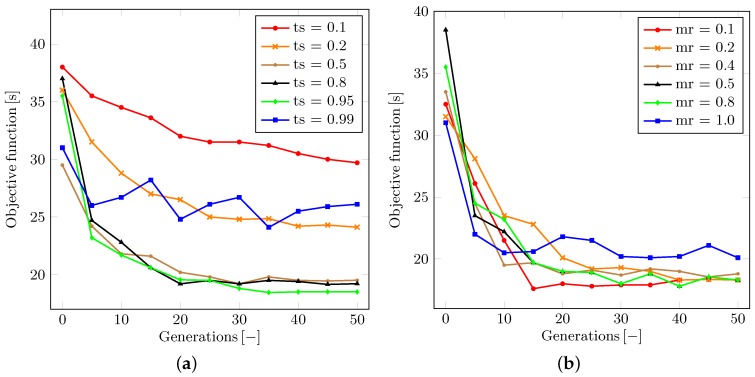
Optimization results for the real traffic scenario. (**a**) Tournament selection ratio; (**b**) Real-life scenario.

We also analyze the behavior pertaining to the tournament selection ratio. Let us denote the tournament selection ratio as ts. The value of ts may be adjusted within the range from zero to one. The ts parameter determines the proportion of population to be removed by the tournament selection at the end of each generation simulation. As shown in Figure 9a, it can be generally argued that the higher the ts value is, the faster the convergence becomes, since more of the weak genomes are removed and replaced by mutations and offspring of stronger survivors. However, if the ts value is close or equal to one, then the solution may become unstable or even divergent. This is because a very low number of strong genomes survive and may eventually lose the tournament selection to other genomes.

To remedy this effect, we may borrow a solution from the simulated annealing [84] or greedy algorithm [88] approaches. However, the ability to achieve the global minimum may still be impaired. We discussed that an impactful parameter is the ratio between the mutation and the interbreeding of genomes while restoring the population back to its maximum size after each tournament selection procedure. Let us denote the ratio in question as mr. Then, as said, the value of this parameter may vary within the range between zero (all new genomes are created as offspring of couples of random surviving genomes) and one (all new genomes are created as mutated clones of a random surviving genome).

To this end, Figure 9b highlights the actual influence of the mr ratio on the convergence of the optimization process. We conclude that the preferred behavior of our solution in the real-life scenario is observed with the mr value set to somewhere between 0.1 and 0.5. This finding corresponds to the processes in nature all around us—a generally more successful approach leads to interbreeding in the population, but a small chance exists that an exceptionally strong genome would be impaired by interbreeding with others.

### 5.4. Performance Evaluation of Employed IoT devices

**Figure 10:**
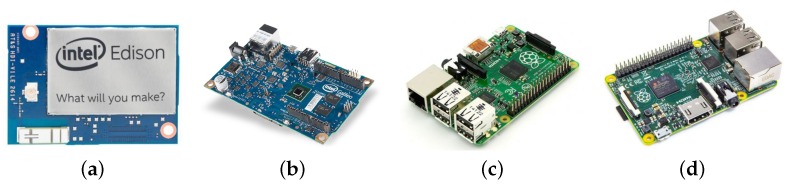
Selected embedded devices. (**a**) Intel^®^ Edison; (**b**) Intel^®^ Galileo Board Gen2; (**c**) Raspberry Pi 1 Model B+; (**d**) Raspberry Pi 2 Model B.

Broadly, the terms “embedded devices” and “embedded systems” refer to electronic technology or computation units that are employed in vehicles, planes, trains, network appliances, etc. Following the fact that the computation performance of this equipment is steadily improving [89] (see Table 3 comparing the hardware parameters of selected devices), the contemporary embedded devices are becoming capable of performing heavy computation tasks similar to the laptop computers. See Figure 10 for details, where selected embedded devices are demonstrated.

In particular, we evaluate the performance of the well-known embedded devices: Intel^®^ Edison, Intel^®^ Galileo board Gen 2, Raspberry Pi (Model B+), and Raspberry Pi 2 (Model B) [90]. Both Raspberry Pi embedded devices run the latest version of the modified operating system Raspbian OS (Jessi, version 8.0) together with the recent version of the Oracle JDK (1.8.0.-b132). Edison and Galileo Gen 2 feature the latest version of the Ubilinux operating system (3.10.17-yoctostandard-r2 build) equipped with the JDK (1.8.0 66-b17)—Ubilinux stands for embedded Linux distribution based on Debian Wheezy and enables to run JVM/JDK.

The target application domains for those platforms are embedded devices with limited memory and storage capacity; the image is currently available for Intel^®^ Galileo Gen 1/Gen2 and Edison. In addition, Edison and Galileo Gen 2 may be attached to a number of different extension boards, for example, to enable the Arduino compatibility. Hence, Edison empowers a range of different use cases, whereas Raspberry Pi might be more suitable for handling graphics as well as supporting multimedia-related applications and products.

To adequately evaluate the performance of the devices listed in Table 3, we implemented the above experiments (see Section 5) as standalone Java applications. In order to run these created experiments on the Raspberry Pi devices, we used Oracle JDK in the terminal. Executing our applications on the Intel^®^ Edison and Galileo Gen 2, we followed the manual to prepare a Linux operating system build equipped with the JRE [91]. Further, the executable *.jar files were designed, deployed, and executed on the devices. To ensure comparable conditions from the memory consumption perspective, the accessible amount of memory was set to 100 MB on all of the devices.

**Figure 11:**
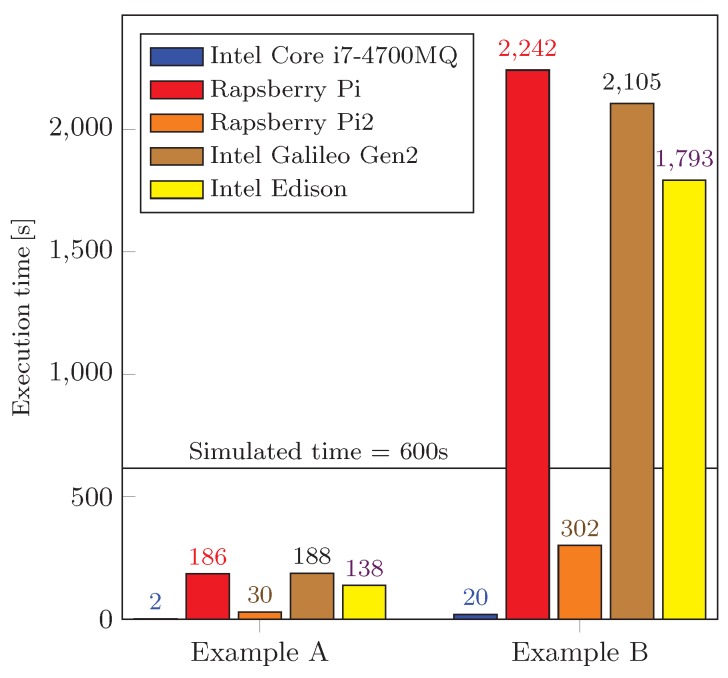
Performance comparison.

The performance evaluation of our tested devices is summarized in Figure 11. We note that there are two groups of devices in terms of their performance. First, the simulation time in each experimental scenario was set to 600 s. The population of genomes was of size 50 and the GA algorithm ran for 50 generations. Based on this, we can conclude that such devices as Intel^®^ Edison, Galileo Gen 2, and Raspberry Pi Model B+ are not capable of offering a sufficient computation performance for the purposes of real-time processing.

On the other hand, Raspberry Pi 2 Model B computes our prepared scenarios several times faster (see Table 4 for a detailed comparison) and therefore becomes a preferred embedded (low-power) device from the performance perspective. If we take a closer look at the difference between the RPi Model B+ and the RPi 2, one could think that the main reason why RPi 2 is almost 7 times faster (for our scenarios) is in the number of CPU cores (single-core vs. quad-core). However, we have implemented our environment as a single thread and hence these performance differences may only be caused by (i) the CPU clock speed (700 MHz vs. 900 MHz) and especially (ii) the change of the SoC architecture (ARM11 vs. Cortex-A7), see Table 3.

Aiming to provide a comprehensive and balanced view, we additionally compared the performance of our tested embedded devices with the laptop Lenovo ThinkPad W540 equipped with the Core i7 4700MQ CPU (Haswell gen.) and having 16 GB of memory. The obtained execution times for the NTB were ten times shorter than those for Rapsberry Pi 2. Noteworthy, Rapsberry Pi 2 is the only embedded device in our experiments having the performance comparable to that of the NTB.

## 6. Conclusions and Lessons Learned

Improving the efficiency of the TMSs (traffic management systems) becomes a hot topic and constitutes a challenging research direction due to a crucial need to monitor the transportation infrastructure in smart cities. This paper first presented various existing technologies usable for traffic data gathering as well as emphasized the emerging communications technologies that are able to significantly improve the accuracy of the collected information. We especially focused on the long-range technologies of today i.e., using the 4G (LTE) cellular networks and Low Power Wide Area (LPWA) solutions, such as SIGFOX and LoRa. We also reviewed some of the many routing protocols in VANET systems ready to disseminate the collected (received) data among vehicles.

Based on the findings from the studied literature, today’s TMS options are still insufficient to construct a reliable and secure TMS capable of meeting the anticipated increase of population as well as numbers of vehicles in smart cities. Therefore, we presented our proposed modular environment for road traffic modeling that allows to easily recreate the examined road infrastructure in its full resemblance. Fueled by the ongoing proliferation of the M2M devices in the IoT domain, the developed environment aims at engaging the embedded (low-power) devices upfront, which plays an important role in our research. To be as close as possible to the real road traffic conditions, our created scenarios follow the structure of the practical urban intersections in Brno (the second largest city in Czech Republic).

For the purposes of our performance evaluation, we developed a unified test environment in Java programing language, where the considered scenarios were implemented. Further, we selected the well-known embedded devices, such as Raspberry Pi 1 (Model B+), Raspberry Pi 2 (Model B), Intel^®^ Edison, and Intel^®^ Galileo Gen 2 for our performance evaluation campaign. Further, we concluded that the preferred embedded device that is comparable (in terms of its performance) with today’s computers is the Raspberry Pi 2 Model B (see Table 4 for the corresponding results). This device therefore becomes an attractive candidate to serve as a computation unit in the realistic TMS deployments.

The main challenge in the modeling and implementation part was to capture the necessary properties of the actual traffic behavior, while having the limited resources of the embedded devices in mind. An event-based discrete dynamic modeling approach was followed, since it constitutes an elegant solution method enjoying the adaptive time step discretization. It is known that the computation time requirements of real-world traffic problems grow rapidly with the increasing complexity of the scenario at hand. However, with the developed solution method, the experienced time-related parameters can be made acceptable.

Together with the solution performance, the behavior of the conducted GA (genetic algorithm) based optimization was studied. It was shown (see Section 5.3) that the GA optimization is convergent and—depending on its parameters—after several generations leads to the desired optimized setup of traffic signals. The monotonicity of this convergence can be further adjusted with the tools coming from the greedy algorithms and the simulated annealing approaches; this task becomes the subject of our future research efforts. Also, the investigation of dynamic routing techniques is of considerable further interest.

Since the traffic model (as well as the smart city itself) should be made aware of the actual congestion levels and feasibility of every route, the traffic flow within a city shall be routed adaptively with respect to the dynamically approximated departure times. Finally, we conclude that the contemporary embedded devices, such as the Raspberry Pi 2 (Model B), already approach the computation power of two-year-old laptops and thus satisfy the requirements to be used as part of the future TMSs in smart cities.

## Figures and Tables

**Table 1:** Connectivity overview for M2M communications/M2M devices [44].

	SIGFOX	LoRa	Clean IoT	NB LTE-M Release 13	LTE-M Release 12/13	EC-GSM Release 13	5G (Targets)
**Range/MCL** **(Maximum Coupling Loss)**	<13 km 160 dB	<11 km 157 dB	<15 km 164 dB	<15 km 164 dB	<11 km 156 dB	<15 km 164 dB	<15 km 164 dB
**Bandwidth**	Unlicensed 900 MHz 100 Hz	Unlicensed 900 MHz <900 Hz	Licensed 7–900 MHz 200 kHz or dedicated	Licensed 7–900 MHz 200 kHz or shared	Licensed 7–900 MHz 1.4 MHz or shared	Licensed 8–900 MHz 2.4 MHz or shared	Licensed 7–900 MHz shared
**Data Rate**	<100 bps	<10 kbps	<50 kbps	<150 kbps	<1 Mbps	<10 kbps	<1 Mbps
**Battery Life**	>10 years	>10 years	>10 years	>10 years	>10 years	>10 years	>10 years
**Availability**	Today	Today	2016/2017	2016/2017	2016/2017	2016/2017	Beyond 2020

**Table 2:** Main characteristics of reviewed vehicular routing protocols.

	Comm. Overhead	Comput. Overhead	Scalability Level	Latency	Delivery Ratio	Network Flexibility	Target Scenario	Infrastructure Dependent
**VADD [64]**	Low	Medium	Medium	Medium	Low	High	Rural	No
**GPCR [65]**	Low	Low	Medium	High	Low	No	Urban	No
**LORA-CBF [66]**	Medium	Low	High	Low	High	Medium	Urban	No
**SADV [67]**	Low	Low	Medium	Medium	Medium	High	Urban	Yes
**UMB [68]**	Medium	Medium	Medium	High	Medium	Medium	Urban	Yes
**ARBR [69]**	Low	Medium	Medium	Medium	High	High	Urban	Yes
**PDGR [70]**	Medium	Medium	Medium	Medium	Medium	No	Urban	No
**MURU [71]**	Low	Medium	Medium	Low	Medium	High	Urban	No
**A-star [72]**	Medium	Low	Medium	Medium	Medium	No	Urban	No
**GyTAR [73]**	Low	Low	Medium	Low	Medium	High	Urban	No
**GVGrid [74]**	Medium	Medium	Medium	Medium	Medium	Medium	Urban	Yes
**BROADCOMM [75]**	High	Low	Medium	Low	Low	Medium	Highway	Yes
**V-TRADE [76]**	Medium	Low	Medium	Medium	Low	No	Highway	No
**IVG [77]**	Low	Low	High	Low	Medium	High	Highway	No
**3rule [78]**	Low	Low	Unknown	Unknown	High	No	All	Yes
**DV-CAST [79]**	Low	Low	High	Low	Medium	Very High	All	No
**CAR [80]**	Medium	Medium	Medium	Medium	Medium	Medium	All	Yes

Used acronyms: **VADD** (Vehicle-Assisted Data Delivery); **GPCR** (Greedy Perimeter Coordinator Routing); **LORA-CBF** (Location-Based Routing Algorithm with Cluster-Based Flooding); **SADV** (Static-node assisted Adaptive data Dissemination protocol); **UMB** (Urban Multi-hop Broadcast protocol); **ARBR** (Adaptive Road-Based Routing); **PDGR** (Predictive Directional Greedy Routing); **MURU** (Multi-Hop Routing Protocol); **GyTAR** (Greedy Traffic Aware Routing protocol); **GVGrid** (A QoS Routing Protocol for Vehicular Ad Hoc Networks); **BROADCOMM** (Emergency Broadcast Protocol for Inter-Vehicle Communications); **V-TRADE** (Vector based TRAck DEtection Protocol); **IVG** (Inter-Vehicles Geocast); **DV-CAST** (Distributed Vehicular BroadCAST); **CAR** (Connectivity-Aware Routing).

**Table 3:** Selected devices and their corresponding specifications.

Device	Type	SoC	Processor	RAM
**Intel**^®^ **Edison**	IoT Development Board	Atom + Quark	500 MHz, Dual-Core Intel^®^ Atom™ CPU, 100 Mhz MCU	1 GB
**Intel**^®^ ** Galileo Gen 2**	IoT Development Board	Quark X1000	400 MHz, Single-Core 32-bit Intel Pentium (ISA)-compatible	256 MB
**Raspberry Pi 1 model B+**	IoT Development Board	BCM2835	700 MHz, Single-Core ARM 11	512 MB
**Raspberry Pi 2 model B**	IoT Development Board	BCM2836	900 MHz, Quad-Core ARM Cortex-A7	1 GB
**Intel**^®^ ** Core i7-4700MQ**	Mobile CPU	Core i7	2.4 GHz, Quad-Core 64-bit support (Haswell architecture)	16 GB

**Table 4:** Comparing results for both scenarios.

		Time [s]	Difference [s]	Ratio [-]
**T-shaped**	Raspberry Pi 1 Model B+	186	156	5.16
	**Raspberry Pi 2 Model B**	**30**	**0**	**1**
	Intel Galileo Gen 2	138	108	4.6
	Intel Edison	188	158	6.26
**Real-life**	Raspberry Pi 1 Model B+	2242	1940	7.42
	**Raspberry Pi 2 Model B**	**302**	**0**	**1**
	Intel Galileo Gen 2	1793	1491	5.93
	Intel Edison	2105	1803	6.97
